# Bacterial community analysis identifies *Klebsiella pneumoniae* as a native symbiotic bacterium in the newborn *Protobothrops mucrosquamatus*

**DOI:** 10.1186/s12866-023-02936-4

**Published:** 2023-08-08

**Authors:** Hung-Yuan Su, Bashir Hussain, Bing-Mu Hsu, Kuo-Hsin Lee, Yan-Chiao Mao, Liao-Chun Chiang, Jung-Sheng Chen

**Affiliations:** 1 Department of Emergency Medicine, E-Da Hospital, I-Shou University, Kaohsiung, Taiwan; 2 School of Chinese Medicine for Post Baccalaureate, I-Shou University, Kaohsiung, Taiwan; 3 Department of Earth and Environmental Sciences, National Chung Cheng University, Chiayi County, Taiwan; 4 Department of Biomedical Sciences, National Chung Cheng University, Chiayi County, Taiwan; 5 School of Medicine, College of Medicine, I-Shou University, Kaohsiung, Taiwan; 6 Department of Emergency Medicine, E-Da Dachang Hospital, I-Shou University, Kaohsiung City, Taiwan; 7 Division of Clinical Toxicology, Department of Emergency Medicine, Taichung Veterans General Hospital, Taichung, Taiwan; 8 School of Medicine, National Defense Medical Centre, Taipei, Taiwan; 9 Department of Medical Research, Taipei Veterans General Hospital, Taipei, Taiwan; 10 Department of Biology and Anatomy, National Defense Medical Centre, Taipei, Taiwan; 11 Department of Medical Research, E-Da Hospital, I-Shou University, Kaohsiung, Taiwan

## Abstract

**Background:**

The study of the native microbiome of organisms is crucial. The connection between the native microbiome and the host affects the formation of the innate immune system and the organism’s growth. However, the native microbiome of newborn venomous snakes has not been reported. Therefore, we aimed to determine the oral and skin microbiomes of newborn *Protobothrops mucrosquamatus.*

**Results:**

We performed 16 S full-length sequencing on 14 samples collected from 7 newborn *P. mucrosquamatus* individuals, specifically targeting their oral and skin microbiomes. In terms of the oral and skin microbiome, the main species were *Klebsiella pneumoniae* lineages. According to subspecies/species analysis, the proportion from highest to lowest was *K. quasipneumoniae* subsp. *similipneumoniae*, *K. pneumoniae* subsp. *pneumoniae*, and *K. pneumoniae* subsp. *rhinoscleromatis*. These three bacteria accounted for 62.5% and 85% of the skin and oral activity, respectively. The oral microbiome of newborn *P. mucrosquamatus* did not comprise common bacteria found in snakebite wounds or oral cultures in adult snakes. Therefore, the source of other microbiomes in the oral cavities of adult snakes may be the environment or prey. Functional Annotation of the Prokaryotic Taxa analysis showed that the skin/oral native microbiome metabolism was related to fermentation and human infection owing to the dominance of *K. pneumoniae* lineages. The characteristics of *K. pneumoniae* may impact the development of venom in venomous snakes.

**Conclusion:**

The results of the native microbiome in the oral cavity and skin of newborn *P. mucrosquamatus* demonstrated that the habitat environment and prey capture may affect the composition of bacteria in adult snakes. We hypothesized that the native microbiome influences newborn venomous snakes and that *K. pneumoniae* lineages related to citrate fermentation may play a role in venom growth. However, further verification of this is required.

**Supplementary Information:**

The online version contains supplementary material available at 10.1186/s12866-023-02936-4.

## Background

With the development of biotechnology, identifying and understanding microbiomes has become a famous field worldwide. In addition to emphasising the human microbiota, other animal or environmental microbiota have also been investigated [1–4]. The interaction between microbiomes and the host is an important research direction, as microbiomes play critical roles in nutrient acquisition, immunity, and disease processes that benefit or harm their hosts [3, 5–8]. Hence, studies on understanding host microbiomes could address many questions associated with the cross-talk between the host and microbial communities, thereby providing many novel findings. The limitations of microbiome studies include time-consuming culture methods and the low efficiency of sequencing technology before the application of next-generation sequencing (NGS) technology [9, 10]. The 16 S ribosomal RNA (rRNA) gene is a powerful tool for identifying bacteria; thus, it is essential to use NGS to harvest 16 S rRNA sequences from samples and classify them with 16 S rRNA microbial inventory approaches for microbiome studies [10]. Third-generation sequencing technologies, such as nanopore or Pacbio single-molecule real-time (SMRT) sequencing, have recently provided high-resolution taxonomic and species-level classification [11, 12]. With increasing studies on microbiomes based on NGS or third-generation sequencing, there is increasing knowledge of the wide microbial taxonomic diversity of environments, humans, mammalian organisms, fish, etc. [6, 13–15]. However, studies on wild non-mammalian vertebrate microbiomes are limited [3].

Squamates are diverse and species-rich animals found in various habitats, ranging from tropical oceans to temperate mountaintops [16]. They have played a critical role in the food chain and ecological recycling; hence, they can be used together with their derivatives to study medical innovations or understand evolutionary biology [3, 17–19]. Most research on the microbiomes of squamate reptiles has concentrated on the gastrointestinal tract of the same host species. These studies revealed gut microbial communities’ evolutionary factors, diversity, and composition [20–22]. However, few studies have investigated squamate reptiles’ oral or skin microbiomes [3, 20–22].

Snakes are one of the most abundant and charismatic squamates worldwide. According to venom characteristics, the snake can be classified as non-venomous or venomous. Snake envenomation causes 20,000 deaths annually in tropical areas. Some patients may develop cellulitis, tissue necrosis, or necrotizing fasciitis complicated by bacterial infection [23–26]. Whether the snakes are venomous or not, bacterial infections after snake envenomation are a significant global public health issue in neglected tropical diseases [27–29]. Six venomous snake species are common in Taiwan, including *Naja atra, P. mucrosquamatus*, *Deinagkistrodon acutus, Trimeresurus stejnegeri, Bungarus multicinctus*, and *Daboia siamensis*, posing a clinical management challenge [24, 30]. Wound infection has been frequently investigated in patients with snakebites, especially venomous snakebites. Further treatment, including antivenom, antibiotics, or surgical intervention, should be performed according to the patient’s condition. Some bacteria, such as *Morganella morganii*, *Enterococcus faecalis*, *Aeromonas spp.*, *Enterobacter spp.*, and *Pseudomonas spp.*, have been frequently identified in surgical wounds. In contrast, *Shewanella spp., Bacteroides fragilis*, *Klebsiella pneumoniae, Proteus spp., Providentia spp., Serratia marcescens*, and *Salmonella spp.* were occasionally identified [24, 26, 31–33]. Antibiotic resistance is another critical issue in snakebite wound infections. For example, *N. atra* envenomation causes the most severe wound infection and the highest surgical rate. The infectious bacterial species abundance was ranked as *M. morganii*, followed by *A. hydrophila* and *Enterococcus spp.* [30, 34]. *M. morganii* isolated from the wound of *N. atra* envenomation was resistant to cefazolin and Augmentin (amoxicillin/clavulanate); therefore, advanced antibiotics (e.g., fluoroquinolone) are required as an effective first-line treatment for patients [34, 35]. Thus, the characteristics and metagenomics of bacteria in snakes provides excellent research and clinical value.

There are diverse bacterial symbioses in a variety of organs in snakes; for example, *Aeromonas* spp., *Salmonella* spp., *Pseudomonas aeruginosa*, and *K. pneumoniae* were the most frequently isolated bacteria in the visceral organs of snakes [28]. Similar to the microbiome in the visceral organs of snakes, oral microbiomes such as *Aeromonas hydrophila, M. morganii*, and *K. pneumoniae* were also commonly found in culture methods [36]. However, using 16 S Sanger sequencing with the culture method, the bacterial species identified only represented 2% of those identified under 16 S metagenomics using NGS [32]. Using 16 S V3-4 metagenomics revealed that the opportunistic pathogens, *Escherichia coli*, *Aeromonas* spp., *Propionibacterium acnes*, *M. morganii*, *Brevibacterium aureum*, *B. fragilis*, and *Shigella* spp., showed high relative abundances in the oral microbiomes of *N. naja, Ophiophagus hannah, Python molurus, Laticauda laticaudata*, *Trimeresurus flavomaculatus*, and *Boiga dendrophila* from the India and Babuyan Island Group [3, 22]. In contrast, another study revealed *Pseudomonas* (62%), *Delftia* (19%), and *Methylobacterium* (9%) as the dominant bacterial genera from oral swabs of *N. atra* in Taiwan [32]. The above-mentioned studies used 16 S V3-4 metagenomic analysis, which is limited to the genus level. Native symbiotic bacterial communities, especially skin and gut bacteria, play a crucial role in sustaining host-microbe symbiosis, neurodevelopment, and immune development [37, 38]. Gut microbiome development during infancy and early childhood affects mental and physical health [39]. With individual growth, the bacterial communities of the individual are changed by the environment and diet. The individual may then face some challenges associated with microbe-host cross-talk, such as cancer [6]. Many studies have shown that probiotics are native symbiotic bacteria from infants or juvenile animals [40]. The skin and oral cavity are the organs where animals first encounter external substances and microorganisms. Therefore, understanding the native microbial communities of the skin and oral cavity can help elucidate the interaction between the immune system and microorganisms, and further explore the effects of native and acquired microbial communities on individuals. This study is the first to investigate the microbiome of newborn snakes. The study aimed to determine the oral and skin microbiomes of newborn *P. mucrosquamatus* snakes and provide a comparison between their oral and skin native symbiotic bacterial communities using 16 S full-length sequencing. By investigating the oral and skin symbiotic bacterial microbiomes of newborn snakes, the study will provide an understanding of the interactions between symbiotic bacteria, applied microbes, and snakes, as well as the ecology or habitat impact on snakes, characteristics of symbiotic bacteria, and snakebite wound infections.

## Materials and methods

### Microbial sampling

Seven hatchlings (newborn snakes) from a live female *P. mucrosquamatus* captured from the wild in the Taipei Basin, with coordinates (25.104949502832916, 121.6283376179647), Taiwan, were used in this study. Once the eggs hatched, the snakelets were temporarily kept in a tank until there was no observable fluid, blood, or other tissue on their bodies. They were then sampled by a professional with sterile water and the animals were immediately released to the Xizhi area of Taipei Basin, where the female *P. mucrosquamatus* was discovered.

Before sampling, commercially available sterile swabs were soaked in a standard saline solution. We then applied the swab to the inside of the hatchling’s cheek and slowly rotated the swab clockwise for 10 s to collect the oral sample. For skin samples, the same method was repeated on the skin’s ventral, lateral, and dorsal sides for 3 s each. These swabs were then placed in a centrifuge tube and kept in a refrigerator at 4 °C before being analysed within 24 h. Each sample was numbered O.AP.003 to O.AP.009 and the corresponding skin sample were numbered S.AP.010 to S.AP.016. The Institutional Animal Ethics Committee of Come Win Biotechnology Ltd. (IACUC22010) approved the experimental protocols. The Taipei City Government approved the use of the snakes.

### Genomic DNA extraction

Genomic DNA (gDNA) extraction from oral and skin swabs was performed using the QIAamp DNA Microbiome Kit (Qiagen, USA). The gDNA extraction was performed according to the manufacturer’s instructions. The concentration of extracted gDNA was determined spectrophotometrically using a NanoDrop 2000 spectrophotometer (Thermo Fisher Scientific Inc., Wilmington, DE, USA). The quality of the extracted gDNA was evaluated by electrophoresis separation (1.5% gel in Tris-acetate ethylenediaminetetraacetic acid buffer), and the purified gDNA was stored at − 20 °C for the 16 S rRNA gene sequencing analysis.

### 16 S rRNA gene sequencing, library construction, and microbial community analysis

Purified gDNA was used to amplify the bacterial full-length 16 S rRNA using a universal primer set (27 F: AGRGTTYGATYMTGGCTCAG and 1492R: RGYTACCTTGTTACGACTT), and the amplicons were sequenced using the PacBio SMRT sequencing platform (Pacific Biosciences Inc., San Diego, CA, USA). The above conditions were used according to the manufacturer’s instructions. Circular consensus sequence reads were obtained from raw PacBio sequencing data using the standard software tools provided by the manufacturer (Pacific Biosciences). The obtained sequences were further analysed using the Quantitative Insights into Microbial Ecology (QIIME2) software package [9] in R (v 4.2.1). The sequence data were trimmed to remove chimeric sequences, marginal sequence errors, and noisy sequences while picking amplicon sequence variants (ASVs) using DADA2 [41] which is also an R package. The taxonomy classification was performed with a 97% threshold limit of similarity against the SILVA database. Additionally, beta diversity was measured based on the Bray–Curtis index, followed by a permutational multivariate analysis of variance (PERMANOVA) using phyloseq [42], and the obtained results were visualized with ggplot2 [43]. The relative abundance of microbes at the genus and species levels was determined using QIIME2 and the obtained results were visualized using ggplot2 and eulerr packages [44]. The distribution pattern of the microbial community based on similarity and dissimilarity was further assessed using statistical analysis of taxonomic and functional profiles [45]. Additionally, linear discriminant analysis effect size (LEfSe) was performed using Galaxy software (http://huttenhower.sph.harvard.edu/lefse/) following Linear discriminant analysis (LDA) score > 2 and *p*-value < 0.05 to identify the differential abundance in microbiota among the experimental groups.

### Microbial functional prediction

The metabolic functions of the microbial communities were predicted using representative sequences and denoised ASV abundance table data. For this purpose, we used the Functional Annotation of the Prokaryotic Taxa (FAPROTAX) pipeline by transforming the ASV table into relevant ecological and metabolic functions associated with the microbial community based on the culture representative of a strain, species, or genus [46, 47]. Furthermore, the significance of the differences in the relative abundance of annotated microbial functions among the experimental groups was analysed using statistical analysis of taxonomic and functional profiles based on a two-tailed Welch t-test (*p* < 0.05), followed by the Benjamini-Hochberg procedure [45]. Pearson correlation analysis was then applied to explore the relationship between the significant enriched taxa and annotated microbial functions, with a *p*-value range of 0.01–0.05. The analysis was performed using IBM SPSS Statistics 24 (IBM, Armonk, North Castle, NY, USA), and the obtained result was visualized using an online software (https://software.broadinstitute.org/morpheus/).

## Results

### Sequence and clustering analysis based on full-length 16 S rRNA sequencing

**Fig. 1:**
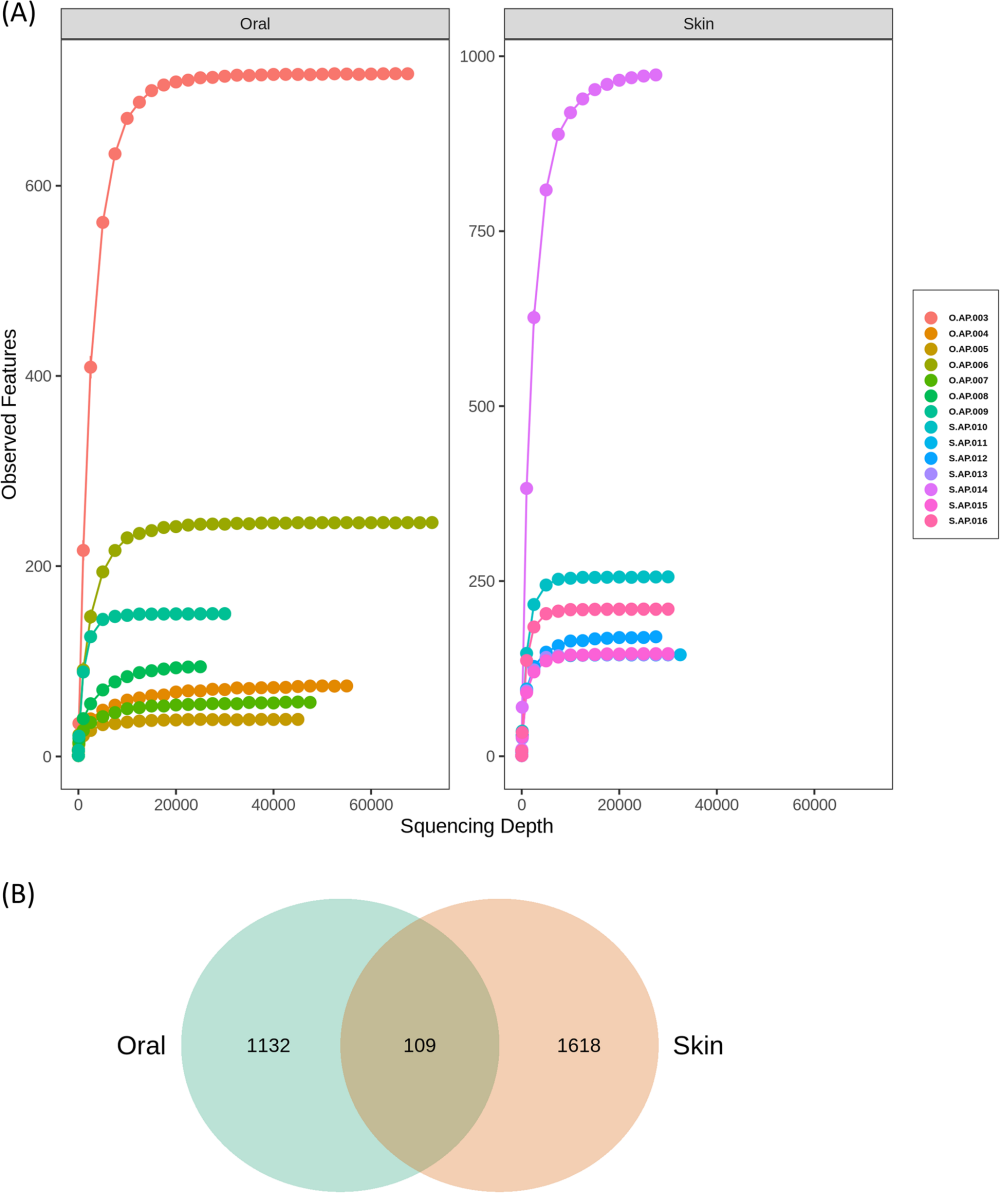
Comparison of rarefaction (**A**) at the lowest sequence depth between the skin and oral microbiota of newborn* Protobothrops mucrosquamatus*. Venn diagram (**B**) representing the shared and unique ASVs between the two experimental groups

 This study obtained a total of 821,031 sequence reads associated with the skin and oral microbiota from seven newborn *P. mucrosquamatus* snakes based on full-length 16 S rRNA sequencing. Among these sequences, 569,106 reads were retained following quality filtration using DADA2, which were later rarefied at a sequence depth of 25,000 to compare the microbial diversity (Fig. 1A), and assigned to 2859 ASVs based on a 97% similarity index using the SILVA database. The rarefaction analysis revealed that the rarefaction curves of all samples approached a saturation plateau, indicating that the sequencing effort was sufficient to estimate microbial richness and diversity at the 97% similarity threshold in the skin and oral microbiota of newborn *P. mucrosquamatus*. According to ASV analysis, skin microbiota showed higher unique features (56.6%) as compared to those of oral microbiota (39.6%), as shown in the Venn diagram (Fig. 1B). However, a small portion of ASVs was shared between the skin and oral microbiota of newborn *P. mucrosquamatus*, indicating a diverse microbial community for the body part. Although the Venn diagram shows a significant difference in microbial communities between the skin and oral cavity, upon inspection of the raw data, it was found that the ASVs with a proportion greater than 1% are similar, while the main difference lies in the ASVs with a proportion less than 1%, where the microbial species on the skin and in the oral cavity are different.

## Oral and skin microbial diversity analysis

**Fig. 2:**
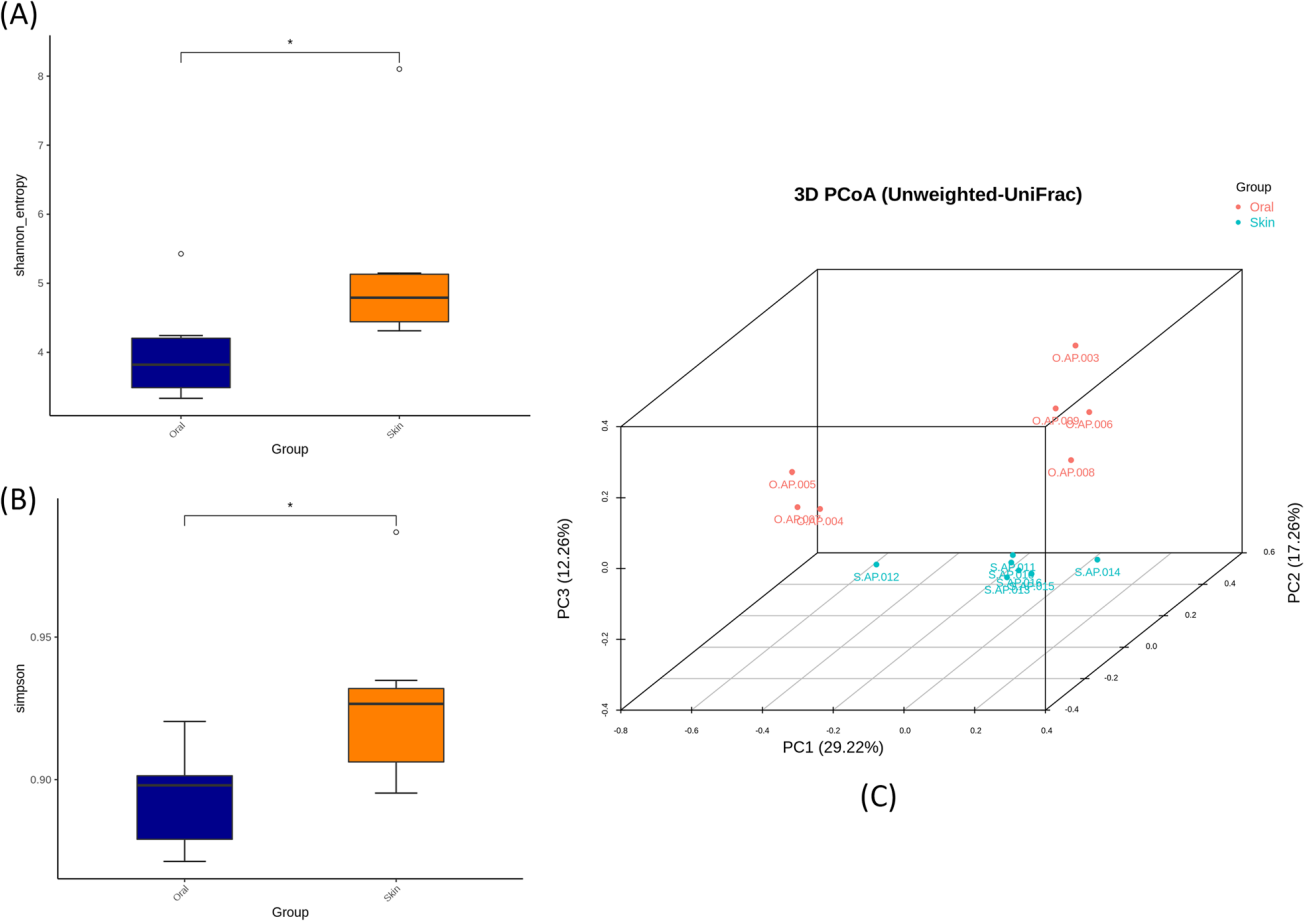
Comparison of bacterial community diversity between the skin and oral cavity of newborn* Protobothrops mucrosquamatus*. Alpha diversity between the oral cavity and skin was measured by (**A**) Shannon and (**B**) Simpson whereas, (**C**) beta diversity based on Bray-Curtis was measured using principal coordinate analysis

The alpha diversity of newborn *P. mucrosquamatus* associated with the oral cavity and skin was evaluated using Shannon (Fig. 2A) and Simpson (Fig. 2B) diversity indices. The alpha diversity indices showed that the skin had higher alpha diversity than that of the oral cavity, which is consistent with the ASVs analysis. The difference in alpha diversity between the oral and skin microbiota of juvenile snakes was statistically significant. Similarly, beta diversity analysis based on Bray-Curtis showed distinct microbiota clustering between the oral cavity and skin, indicating a higher degree of dissimilarity in beta diversity, as shown in Fig. 2C. Furthermore, the adonis function based on PERMANOVA revealed that the difference in beta diversity was statistically significant between the oral cavity and skin of newborn *P. mucrosquamatus* (*p* < 0.05).

### Oral and skin microbial community composition and abundance analysis

**Fig. 3:**
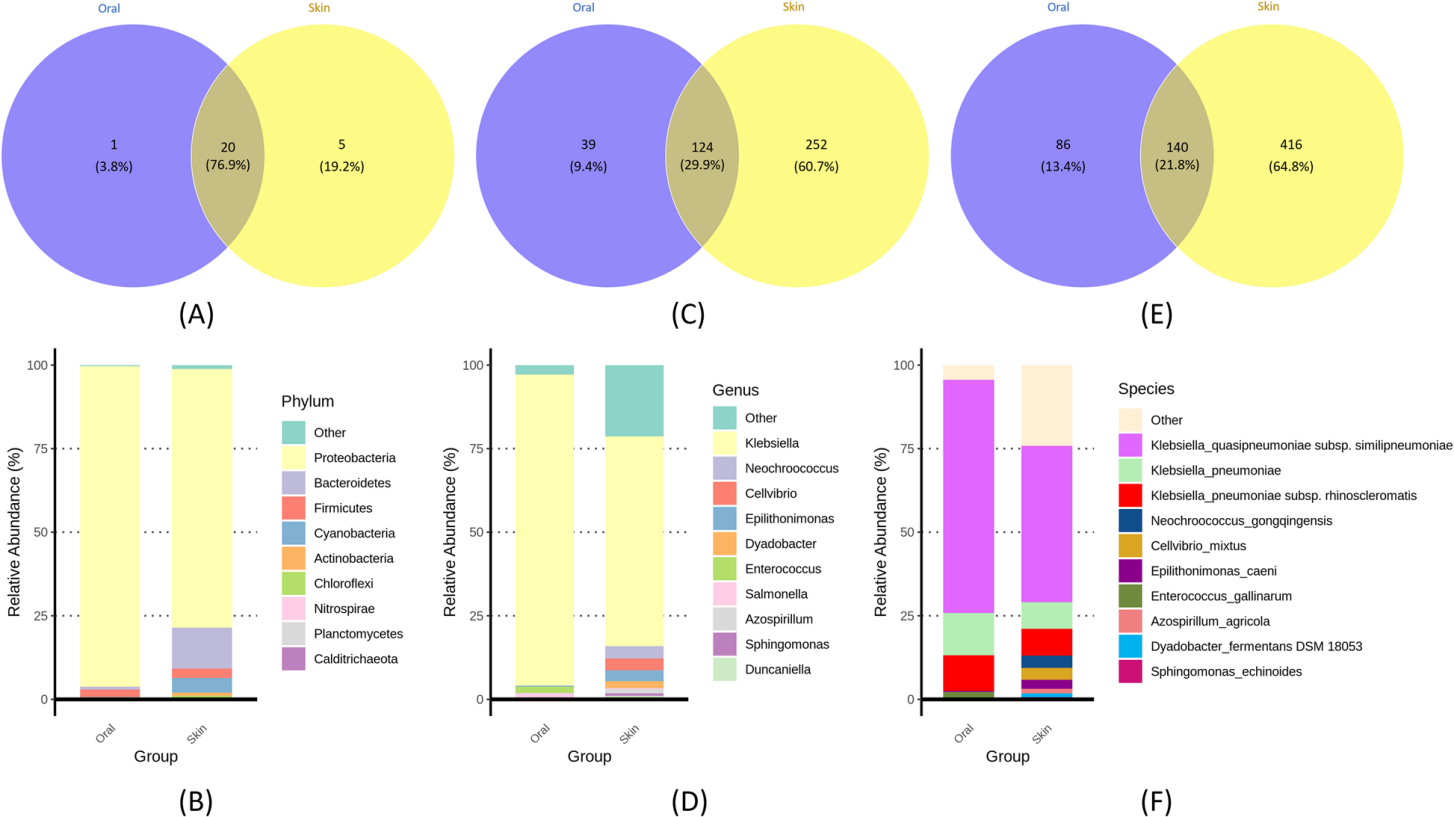
Bacterial taxonomic composition and abundance at phylum, genus and species levels associated with newborn* Protobothrops mucrosquamatus* oral cavity and skin. Venn diagrams showing the shared and unique Taxa between the oral cavity and skin

Taxonomic analysis based on full-length 16 S rRNA revealed striking differences in the composition and abundance of the microbial community at the taxonomic level between the oral cavity and skin of newborn *P. mucrosquamatus*. A total of 26 phyla, 415 genera, and 642 species were associated with the oral cavity and skin of newborn *P. mucrosquamatus*. Among the 26 phyla, five unique phyla were associated with the skin (19.2%). In contrast, only one phylum was associated with the oral cavity (3.8%), as shown in Fig. 3A. However, a large proportion of phyla were common (76.9%) between the oral cavity and skin, indicating a higher similarity at the phylum level. The relative abundance of the top ten phyla associated with the oral cavity and skin of newborn *P. mucrosquamatus* are shown in Fig. 3B). Proteobacteria was the most common phylum in the skin and oral cavity. The second-most abundant phylum in the skin was Bacteroidetes, whereas Firmicutes was the second-most abundant phylum in the oral microbiota. At the genus level, 415 genera and 642 species were obtained, with striking differences in composition and abundance between the oral and skin microbiota. Among them, 29.9% of genera and 21.8% of species were common in the oral cavity and skin (Fig. 3C and E). However, a large proportion of the genera (60.7%) was observed in the skin, whereas the remaining 9.4% of genera were associated with the oral microbiota, indicating that the skin microbiota is more diverse compared to that of the oral microbiota at the genus level, which is consistent with ASVs and diversity analysis. *Klebsiella* was the most dominant genus in the oral cavity and skin (Fig. 3D). *K. pneumoniae* lineages were discussed as critical species in the oral cavity and skin by species-level analysis (Fig. 3F). The second-most abundant genus in the oral cavity group was *Neochroococcus*, whereas *Enterococcus* was present in the oral cavity. Taxonomic analysis revealed a total of 642 species with a striking difference in composition and abundance, similar to the genus-level comparison between the oral cavity and skin of newborn *P. mucrosquamatus*. The relative abundance of each sample is shown in Fig. 3. For oral samples, the bacterial composition of O.AP.008 was different from others; for skin samples, the bacterial composition of S.AP.011, S.AP.014, and S.AP.016 were different from others, especially *Neochroococcus gongqingensis*, which was rich in S.AP.011. In brief, bacterial diversity was higher in the skin samples than that in the oral samples.

Furthermore, we performed Principal Component Analysis (PCA) to determine the variation in the bacterial community based on composition and abundance at the species level. PCA analysis showed a distinct clustering of these species based on their uniqueness and distribution in the oral cavity and skin of newborn *P. mucrosquamatus*, as shown in Fig. S1. At the genus level, only 21.8% of genera were common between the oral cavity and skin (Fig. 3E). However, 64.8% of genera were associated with the skin. In contrast, only 13.4% of genera were associated with oral bacteria, indicating a more diverse microbial community associated with skin than that associated with the oral cavity.

### Differential analysis at taxonomic levels associated with the oral cavity and skin

**Fig. 4:**
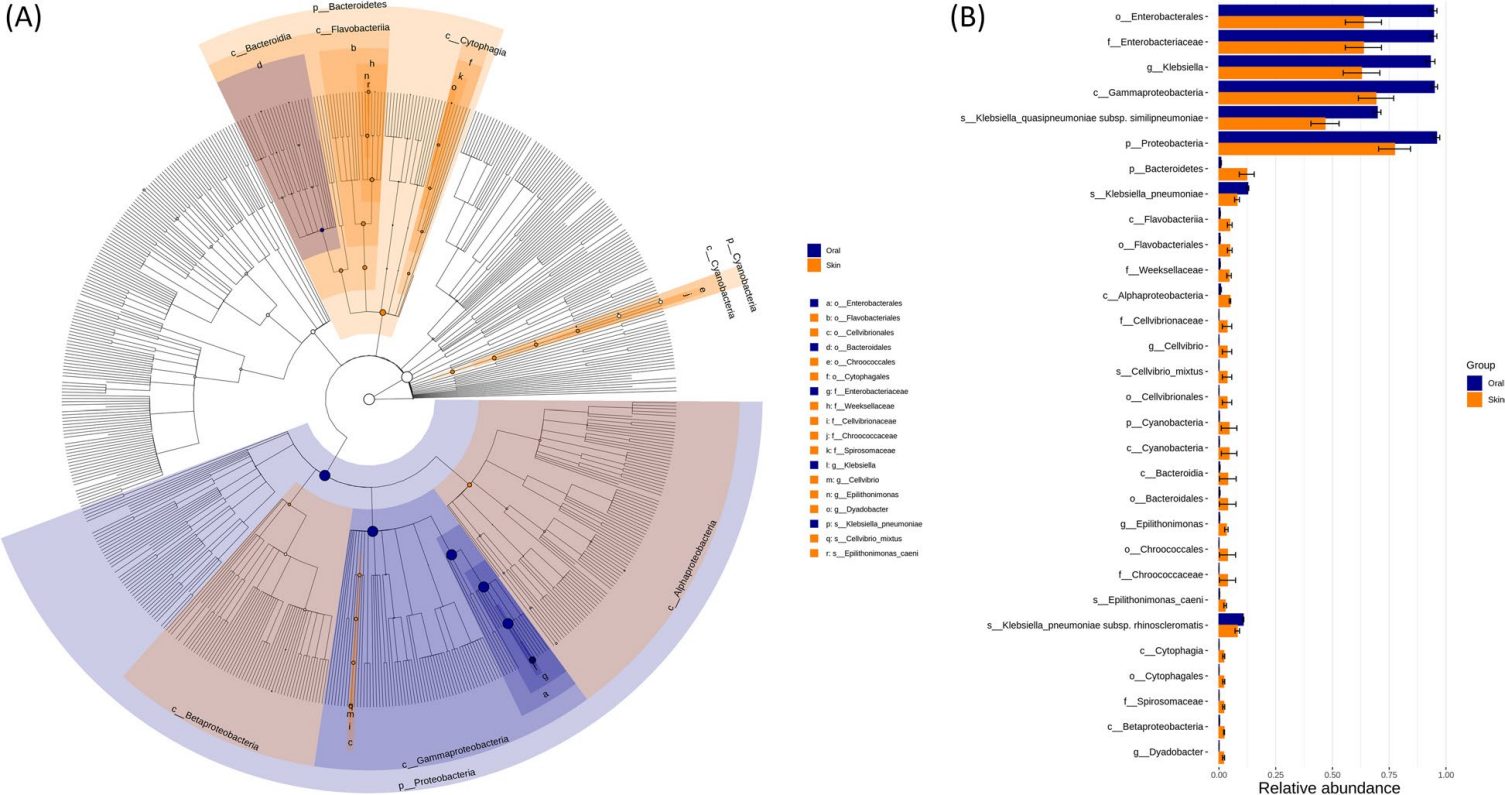
Cladogram (**A**) showing the differentially abundant taxa at the species level associated with oral and skin microbiota of newborn* Protobothrops mucrosquamatus*. The bar graph (**B**) showing the relative abundance of these differential abundant taxa associated with the oral cavity and skin of newborn* Protobothrops mucrosquamatus*

LEfSe analysis coupled with a logarithmic LDA score analysis was performed to explore the statistically differential bacterial taxa between the oral and skin microbiota of newborn *P. mucrosquamatus* as shown in Fig. 4A. LEfSe analysis revealed 30 statistically differential bacteria at different taxonomic levels. Among them, 21 significant bacteria were enriched in the skin, whereas only nine were statistically enriched in the oral environment. Additionally, these significantly enriched bacteria associated with the skin were higher in abundance than oral bacteria, as shown in Fig. 4B, which shows species with significant differences between groups.

### Microbial functional analysis associated with the oral cavity and skin

Functional annotation analysis based on the FAPROTAX pipeline revealed 73 ecological and metabolic functions associated with the oral cavity and skin microbiota of newborn *P. mucrosquamatus*. We performed PCA analysis to further determine the variation in these annotated functions based on composition and abundance associated with the oral cavity and skin, as shown in Fig. S2. PCA analysis showed distinct clustering of these annotated functions based on their uniqueness and distribution in the oral cavity and skin of newborn *P. mucrosquamatus*.

**Fig. 5:**
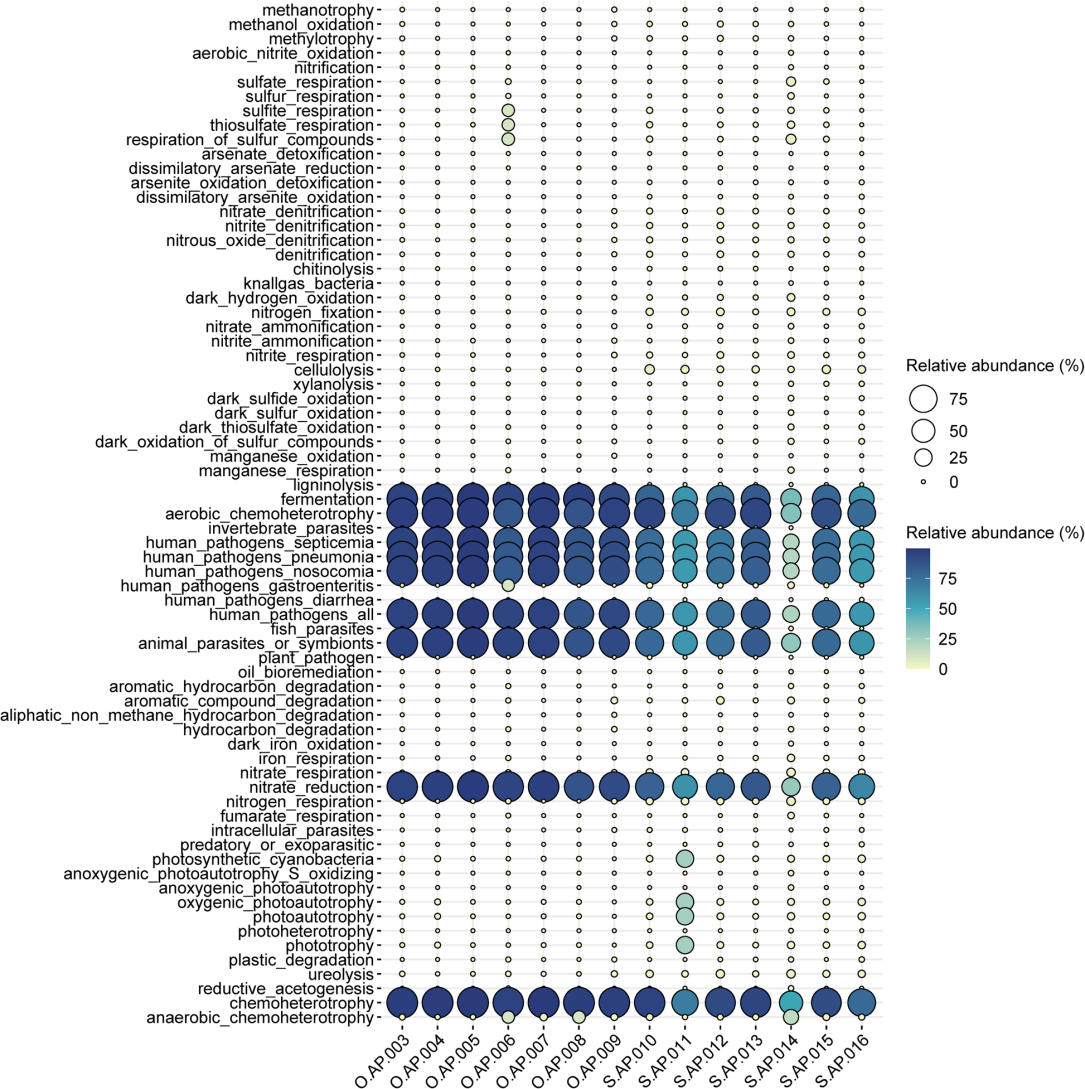
Annotated ecological and metabolic functions of bacterial community associated with the oral cavity and skin of newborn* Protobothrops mucrosquamatus* based on FAPROTAX pipeline. Bubble plot showing the relative abundance (%) of these functions associated with the oral cavity and skin of newborn* Protobothrops mucrosquamatus*

The abundance and composition of these annotated microbial functions are presented in the form of a bubble plot in Fig. 5. The majority of these annotated microbial functions, including chemoheterotrophy, nitrate reduction, fermentation, aerobic chemoheterotrophy, human pathogens, and animal symbionts, were more abundant in the oral cavity than those in the skin.

Furthermore, we performed statistical analysis using STAMP software to explore the significant enrichment of these annotated microbial functions in the oral cavity and skin of newborn *P. mucrosquamatus*. Comparative statistical analysis revealed that three annotated microbial functions, including animal parasites or symbionts, fermentation, and human pathogens, were significantly enriched (*p* < 0.05) in the oral cavity with a higher abundance than that in the skin. However, the remaining five annotated functions: cellulolysis, nitrate respiration, nitrogen fixation, nitrogen respiration, and ureolysis, were significantly enriched (*p* < 0.05) in the skin with a higher abundance than that in the oral environment.

### Association between the annotated functions and microbial community

**Fig. 6:**
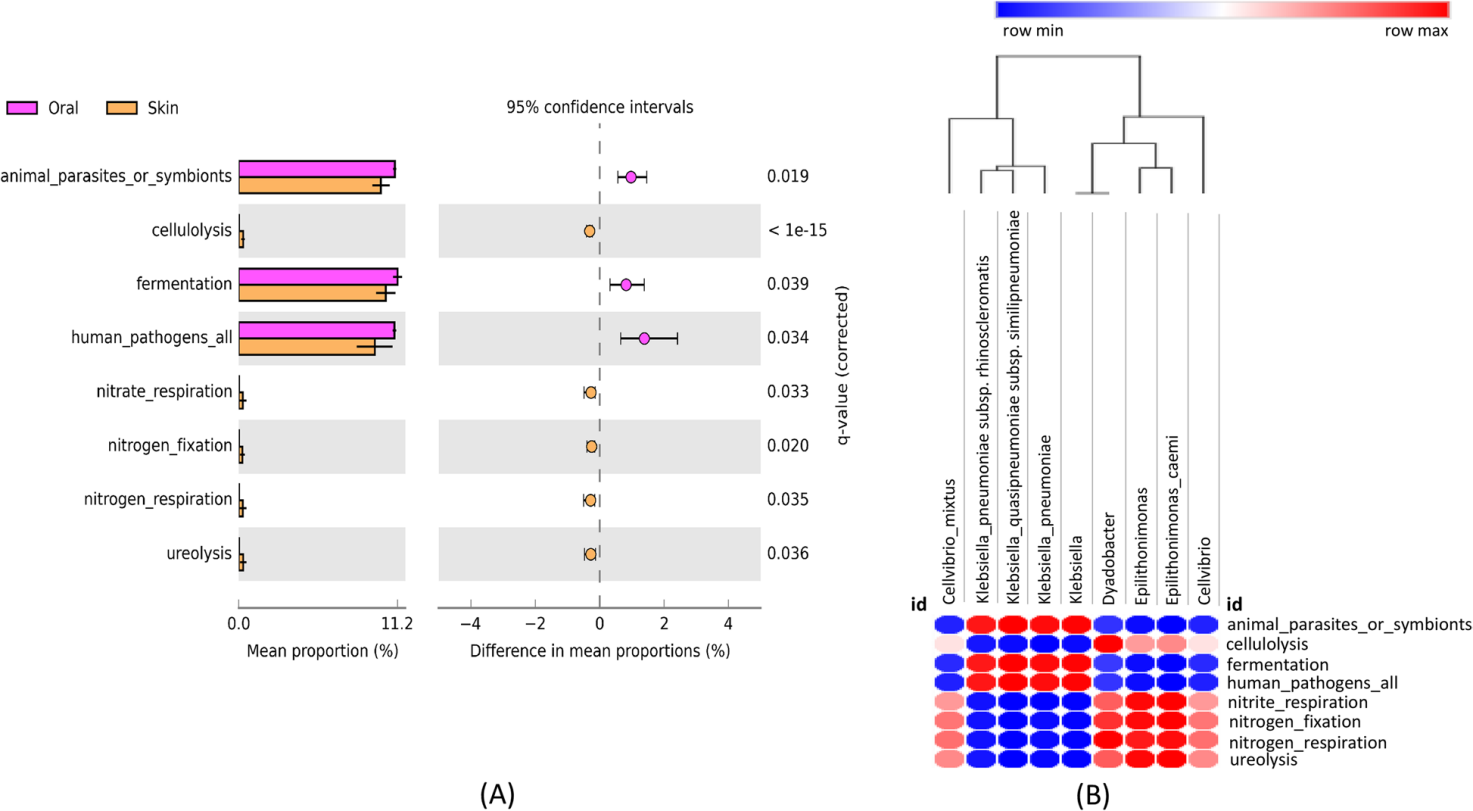
The post hoc plot (**A**) of enriched microbial predicted functions associated with the oral cavity and skin of newborn* Protobothrops mucrosquamatus*. The left panel of these figures shows the abundance ratio of differentially enriched Kyoto Encyclopedia of Genes and Genomes (KEGG) pathways. The right panel represents the significant difference at *p* < 0.05, whereas the middle one indicates the mean proportion of differentially enriched KEGG pathways in the 95% confidence interval. Pearson correlation analysis between the bacterial gut community at the species level and predicted pathways based on full-length 16S rRNA sequencing (**B**). The positive and negative correlations are indicated in red and green colours

Pearson correlation analysis was applied to further determine the contribution of the significantly enriched bacteria at the genus and species levels to the significantly enriched annotated microbial functions associated with the oral cavity and skin (Fig. 6A and B). The correlation analysis revealed that *Klebsiella* at the genus, species, and subspecies levels had a significant positive correlation (*p* < 0.01) with human pathogens, animal parasites or symbionts, and fermentation. In contrast, it had a significant negative correlation (*p* < 0.01) with cellulolysis, nitrite respiration, nitrogen fixation, nitrogen respiration, and ureolysis. Conversely, *Epilithonimonas* at the genus and species levels had a significant positive relationship with cellulolysis (*p* < 0.05), nitrite respiration (*p* < 0.05), nitrogen fixation (*p* < 0.01), and ureolysis (*p* < 0.01). In contrast, it was significantly negatively correlated with animal parasites or symbionts (*p* < 0.05) and fermentation (*p* < 0.05). Additionally, *Dyadobacter* at the genus level was significantly positively correlated with cellulolysis (*p* < 0.01), nitrogen fixation (*p* < 0.05), and nitrogen respiration (*p* < 0.05). However, *Cellvibrio* at the genus and species levels had an insignificant correlation with any annotated microbial functions.

## Discussion

The native microbiome plays a critical role in the innate immune response after birth. Few native microbiomes are considered probiotics and have been used in various applications [48–50]. Investigating the native microbiome of various organisms is important for understanding the role of microorganisms in their early growth stages. Food ingestion and contact with wildlife can provide newborn organisms with various microorganisms that can act as commensal microorganisms. This study describes the native oral and skin microbiomes of newborn *P. mucrosquamatus* without food ingestion or environmental contamination. This is the first study to investigate the microbiota in newborn snakes.

Most snake oral bacterial flora studies have relied on culture methods [51, 52]. A local study revealed that the dominant isolated bacterial species from the oral cavity of adult *P. mucrosquamatus* in Taiwan were *P. aeruginosa*, *B. fragilis*, *Clostridium* spp., *Proteus vulgaris*, and *E. faecalis*, whereas *K. pneumoniae* accounted for only 16.7% [51]. *Bacteroides spp., Clostridium spp., Enterococcus spp.*, *Pseudomonas spp.*, and *Acinetobacter sp.* were the dominant bacterial species found in *P. mucrosquamatus* bite wound infections [53, 54]. The oral microbiomes of *P. mucrosquamatus* newborns in our study differed from those of adults and biting wound infections, indicating that the acquired environment and diet significantly impact microbiome changes.

Skin microbiomes are critical for protection against infection and maintaining health [55]. However, few studies have focused on the reptilian skin microbiome, and most have focused on the pathogenic microorganisms of reptile skin diseases [55, 56]. A study indicated that the skin microbiome of komodo dragons (*Varanus komodoensis*) has a higher microbial diversity than the oral microbiome, which agrees with our findings [21]. Fungal dermatitis is a common snake disease that shifts the skin microbiome [57, 58]. The protective microbiome is a critical concern, and the native microbiome is a key factor [59, 60]. Our results serve as a reference for the prevention of skin diseases in snakes.

Previous studies have shown significant differences between oral and skin microbiomes in reptiles and snakes [55]. However, no such differences were found in the newborn *P. mucrosquamatus*; the predominant bacterial communities found in both the skin and oral cavity of newborn snakes in this study were similar. However, there are greater relative differences in the ASVs that comprise less than 1% of the skin microbiome when compared to the oral cavity. Additionally, these ASVs have more homology with environmental microbes, indicating that the skin microbiome of newborn snakes is more susceptible to environmental influences. The similarity of the dominant bacterial communities suggests that these microbes are native to *P. mucrosquamatus*. Consequently, further analysing the interior or exterior of the eggshell can help understand the sources of the native microbial species in the current study. Additionally, the diverse bacterial communities found in adult snakes from different countries, combined with our results, suggests an adaptive microbiome dependent on habitat, thereby aligning with the findings from a study on Komodo dragons [21]. Unfortunately, to date, no studies have characterised newborn snake skin or oral microbial communities; hence, there is no existing knowledge to compare.

In this study, *K. pneumoniae* lineages (*K. pneumoniae* and *K. quasipneumoniae*) were dominant in the oral and skin microbiomes of newborn *P. mucrosquamatus*, suggesting a key role in the snake’s innate immune system. Therefore, it will be helpful to understand the interaction between native bacteria and the host by analysing the genomic and proteomic features of *Klebsiella* spp. isolated from newborn snakes. Moreover, *K. pneumoniae* is responsible for many hospital-acquired infections and is one of the most critical multidrug-resistant microorganisms worldwide [61, 62]. Because *K. pneumoniae* can be found in various environments and animals, it has been considered a public health issue and requires urgent to monitoring [63]. *Klebsiella pneumoniae* has been previously subdivided into three taxonomic subspecies, *K. pneumoniae* subsp. *pneumoniae, K. pneumoniae* subsp. *rhinoscleromatis* and *K. pneumoniae* subsp. *ozaenae* based on the KpI phylogenetic tree [64]. With the development of molecular technology, an increasing number of subgroups have been reported, such as *K. quasipneumoniae*, which is a novel lineage of *K. pneumoniae* [65]. *Klebsiella pneumoniae* and *K. quasipneumoniae* are zoonotic pathogens with severe antibiotic resistance [66].

*Klebsiella* spp. play a significant role in the skin and oral microbiome of newborn *P. mucrosquamatus* snakes. Results from the microbial function predictions indicated high fermentation activities of *K. pneumoniae* and *K. quasipneumoniae*. Snakes use their bodies to capture prey and potentially transfer *K. pneumoniae* lineages into their digestive systems after swallowing. Many studies have indicated that *K. pneumoniae* possesses diverse citrate fermentation genes and participates in citrate fermentation [67–69]. Citrate is responsible for 2.3–12.9% of various snake venoms and inhibits snake venom proteases [70]. This study raises the hypothesis that native *K. pneumoniae* or its lineage may play a crucial role in venom development in newborn venomous snakes; however, further research is needed to confirm this hypothesis. Therefore, isolating *K. pneumoniae* lineages from newborn venomous snakes is an important task to gain further insight into the interactions between the host and microbiome in snakes.

## Conclusion

*Klebsiella pneumoniae* lineages play a crucial role in the skin and oral microbiome of newborn *P. mucrosquamatus*, accounting for more than 60% of the microbiome. A limitation of this study is the lack of isolation of *K. pneumoniae* from the samples. Although the oral microbiome is consistent among individuals, the skin microbiome varies. This study proposed two essential points. First, adult snake oral and skin microbiome composition is affected by the external environment or diet, resulting in soil-associated bacteria and *Enterobacter* being dominant. Thus, snakebite-related infections may be associated with the habitat and environment. Second, our study hypothesises that *K. pneumoniae* lineages may play a role in venom production and maturation during venomous snake development. This study serves as an essential reference for future research on venomous snakes.

### Supplementary Information

**Additional file 1: Fig. S1.** PCA plot showing the distinct clustering in the bacterial diversity at the genus level in the oral cavity and skin. **Fig. S2.** PCA plot showing the distinct clustering in the annotated microbial functions using FAPROTAX in the oral cavity and skin of newborn* Protobothrops mucrosquamatus*.

## Data Availability

The datasets generated and/or analysed during the current study are available in the [Figshare] and [NIBI] repositories, [10.6084/m9.figshare.23551629] and [PRJNA990549]. The samples were assigned consecutive accession numbers ranging from SAMN36268686 to SAMN36268699, while the raw sequencing data were assigned consecutive numbers from SRR25114049 to SRR25114062 in NCBI.

## Abbreviations

FAPROTAX: Functional Annotation of the Prokaryotic Taxa
NGS: Next Generation Sequencing
PERMANOVA: Permutational multivariate analysis of variance
rRNA: Ribosomal RNA
gDNA: Genomic DNA
QIIME2: Quantitative Insights into Microbial Ecology
ASVs: Amplicon sequence variants
LEfSe: Linear discriminant analysis effect size
