# Supplementary material for: Bacterial community analysis identifies Klebsiella pneumoniae as a native symbiotic bacterium in the newborn Protobothrops mucrosquamatus
Source: BMC Microbiol. 2023 Aug 8;23:213. doi: 10.1186/s12866-023-02936-4 (PMC10408043; doi:10.1186/s12866-023-02936-4)
Supplement: Supplementary file 1 — Additional file 1: Fig. S1. PCA plot showing the distinct clustering in the bacterial diversity at the genus level in the oral cavity and skin. Fig. S2. PCA plot showing the distinct clustering in the annotated microbial functions using FAPROTAX in the oral cavity and skin of newborn Protobothrops mucrosquamatus. [file 12866_2023_2936_MOESM1_ESM.docx]

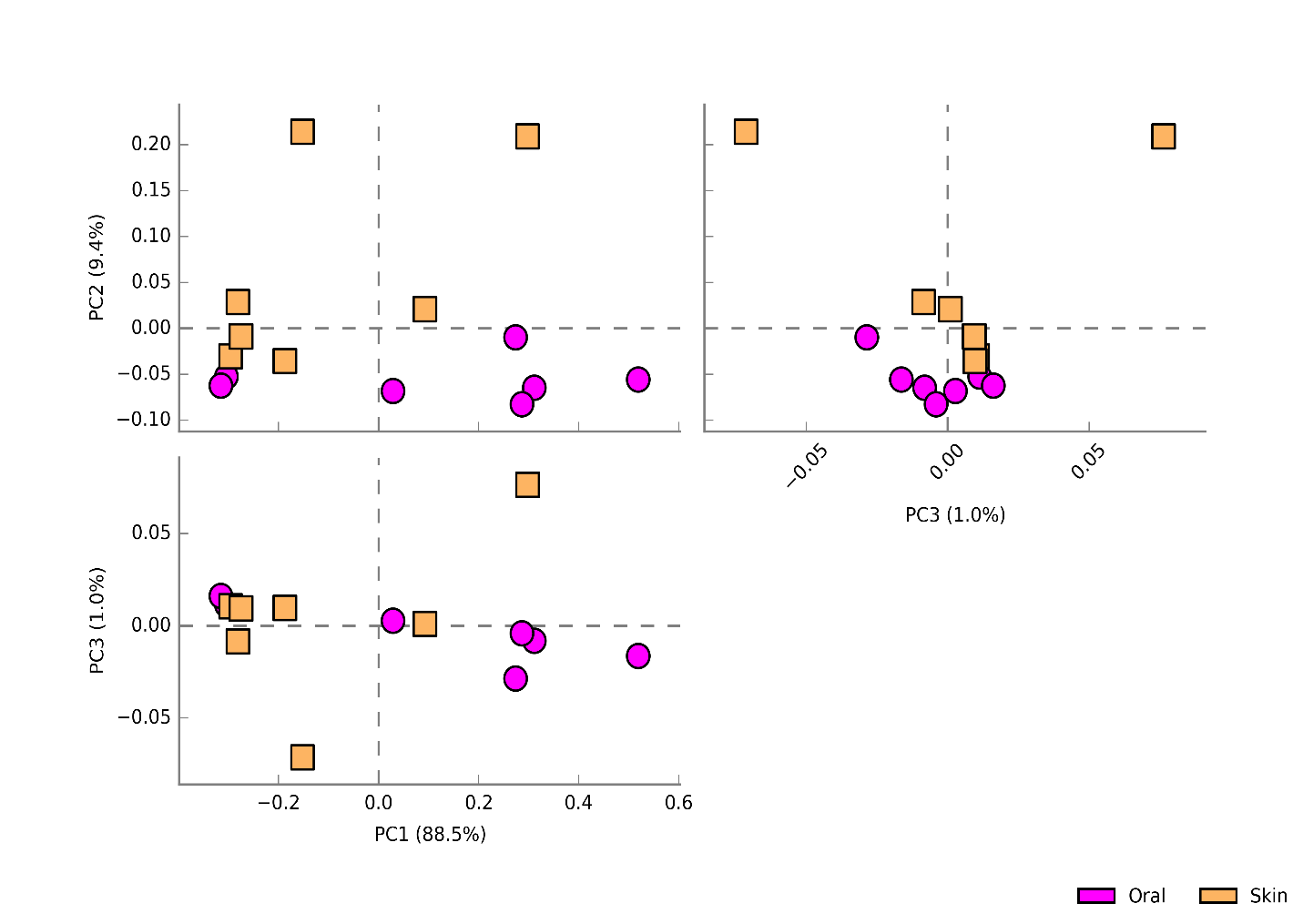


Fig. S1. PCA plot showing the distinct clustering in the bacterial diversity at the genus level in the oral cavity and skin.


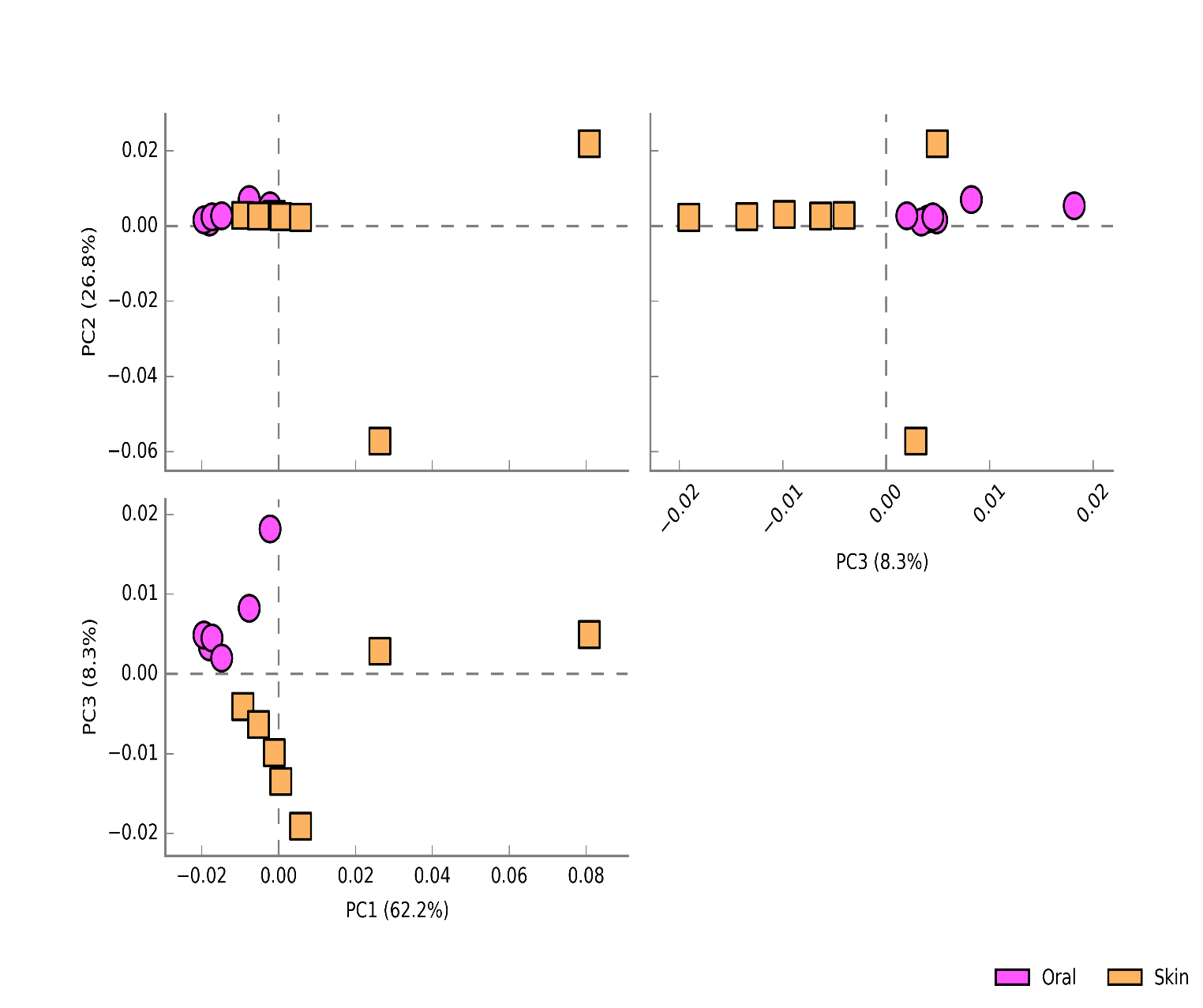


Fig. S2. PCA plot showing the distinct clustering in the annotated microbial functions using FAPROTAX in the oral cavity and skin of newborn *Protobothrops mucrosquamatus*.
